# Supplementary figures and images for: Thiamethoxam exposure deregulates short ORF gene expression in the honey bee and compromises immune response to bacteria
Source: Sci Rep. 2021 Jan 15;11:1489. doi: 10.1038/s41598-020-80620-7 (PMC7811001; doi:10.1038/s41598-020-80620-7)

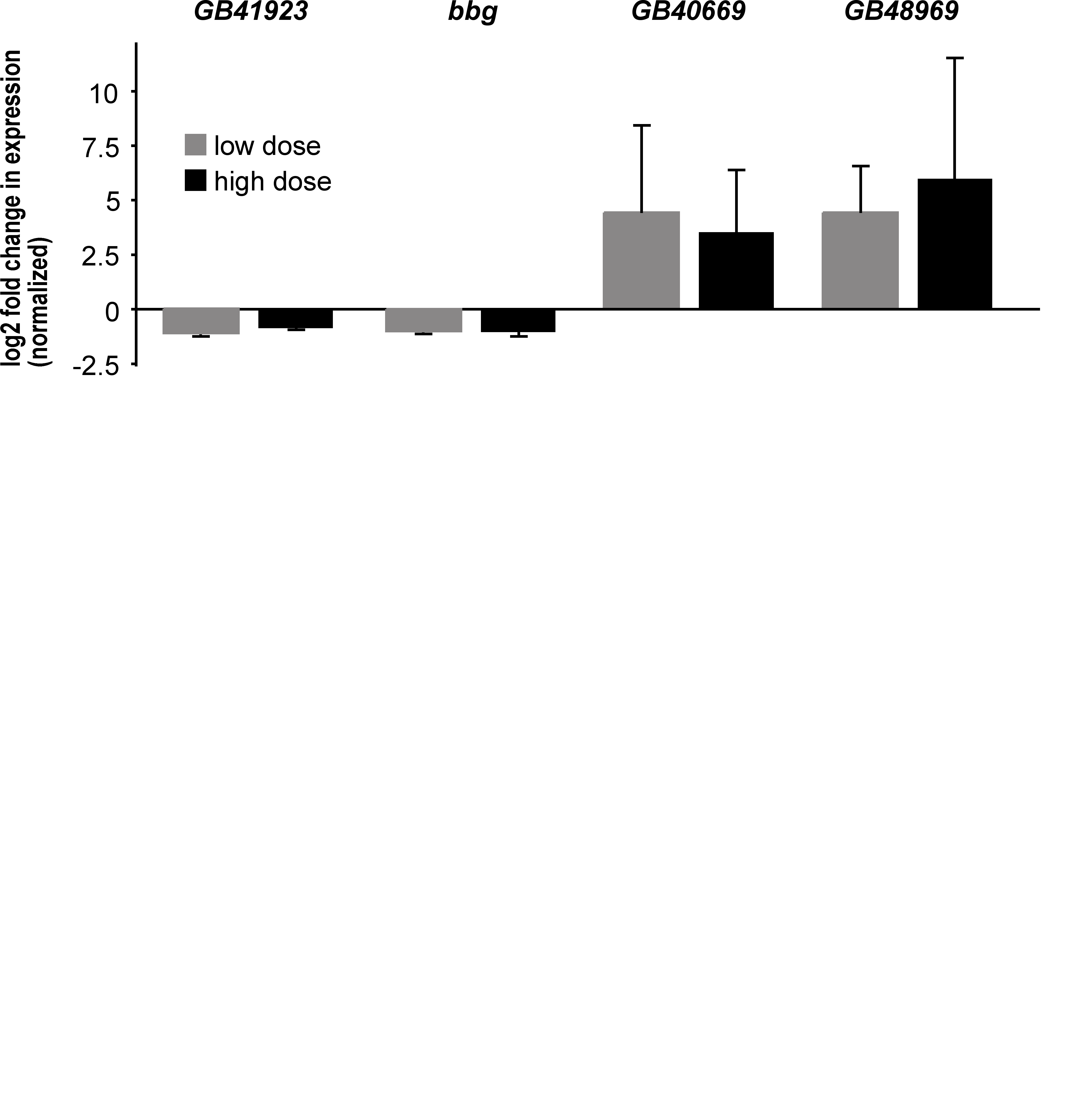

Supplement: Supplementary file 1 — Supplementary Information 1. [file 41598_2020_80620_MOESM1_ESM.tif]
