# Supplementary material for: Thiamethoxam exposure deregulates short ORF gene expression in the honey bee and compromises immune response to bacteria
Source: Sci Rep. 2021 Jan 15;11:1489. doi: 10.1038/s41598-020-80620-7 (PMC7811001; doi:10.1038/s41598-020-80620-7)
Supplement: Supplementary file 4 — Supplementary Information 4. [file 41598_2020_80620_MOESM4_ESM.docx]

- **Thiamethoxam exposure deregulates short ORF gene expression in the honey bee and compromises immune response to bacteria**
- Pâmela Decio1*, Pinar Ustaoglu2*, Kamila Derecka3, Ian C. W. Hardy3, Thaisa C. Roat1, Osmar Malaspina1, Nigel Mongan4, Reinhard Stöger3, 5 and Matthias Soller2, 5
- 1Universidade Estadual Paulista (UNESP), Instituto de Biociências, Campus Rio Claro, São Paulo, Brazil.
- 2School of Biosciences, College of Life and Environmental Sciences, University of Birmingham, Edgbaston, Birmingham, B15 2TT, United Kingdom
- 3School of Biosciences, University of Nottingham, LE12 5RD, United Kingdom
- 4School of School of Veterinary Medicine and Science, University of Nottingham, LE12 5RD, United Kingdom
- **Running title**: Thiamethoxam exposure, short ORFs and bee immunity
- **Key Words**: Neonicotinoid, *Apis mellifera*, anti-microbial peptides,
- 5Corresponding authors: [m.soller@bham.ac.uk](mailto:m.soller@bham.ac.uk), [reinhard.stoger@nottingham.ac.uk](mailto:reinhard.stoger@nottingham.ac.uk)
- *equally contributing authors

**Supplemental information and discussion**

- **Analysis of alternative splicing**
- As alternative splicing has been suggested as a mechanism to adapt gene expression to environmental changes ^15,44^, we analysed the RNA-seq data for changes in alternative splicing. We found significant differences (Supplemental Data 2). Subsequent inspection of sequencing traces using Integrated Genome Viewer (IGV) ^41^ found that most of these genes had complex splicing patterns that showed no obvious differences in the number of sequence reads over alternatively spliced gene sections (data not shown); therefore, we did not explore these genes further in this study.
- Not to find changes in alternative splicing in response to low dose long-term exposure was unexpected, but we also did not find changes upon acute high dose xenobiotic exposure in selected genes in our earlier study on bees ^17^. In other insects, alternative splicing of the *Dscam* gene, which acts as a pattern recognition receptor in the immune response, can generate many different isoforms ^68,69^ and the splicing patterns can change upon bacterial infection ^70^. Since *Dscam* alternative splicing is robust against perturbations of splicing factors ^71,72^, the absence of changes might indicate that a specific immune challenge is required to change its splicing pattern ^70^. We previously also analysed alternative splicing in bee *elav* and *Xbp-1* genes as potential markers for defects in synapse formation and the stress response upon high dose acute thiamethoxam exposure, but also low dose chronic exposure did not affect their splicing ^17,52,53^. In contrast to most other species, bees have only a single *elav* gene making it unlikely that the lack of splicing differences is due to redundancy among close related ELAV RNA binding proteins ^73^. Potentially, alternative splicing might be changed only in a few cells in the brain, eluding detection without single cell analysis, or could in addition also be more subtle, requiring more replicates for detection of significantly altered alternative splicing changes ^15^.

**References**

83. Hemani, Y. & Soller, M. Mechanisms of Drosophila Dscam mutually exclusive splicing regulation. *Biochem. Soc. Trans.* **40**, 804–809 (2012).

84. Schmucker, D. *et al.* Drosophila Dscam is an axon guidance receptor exhibiting extraordinary molecular diversity. *Cell* **101**, 671–84 (2000).

85. Dong, Y., Taylor, H. E. & Dimopoulos, G. AgDscam, a Hypervariable Immunoglobulin Domain-Containing Receptor of the Anopheles gambiae Innate Immune System. *PLoS Biol.* **4**, e229 (2006).

86. Ustaoglu, P. *et al.* Srrm234, but not canonical SR and hnRNP proteins, drive inclusion of *Dscam* exon 9 variable exons. *RNA* **25**, 1353–1365 (2019).

87. Haussmann, I. U. *et al.* Plasmid-based gap-repair recombineered transgenes reveal a central role for introns in mutually exclusive alternative splicing in Down Syndrome Cell Adhesion Molecule exon 4. *Nucleic Acids Res.* **47**, 1389–1403 (2018).

88. Zaharieva, E., Haussmann, I. U., Bräuer, U. & Soller, M. Concentration and Localization of Coexpressed ELAV/Hu Proteins Control Specificity of mRNA Processing. *Mol. Cell. Biol.* **35**, 3104–15 (2015).

**Supplemental Figure legend**

- **Supplemental Figure 1: Validation of thiamethoxam induced differentially expressed genes by RT-qPCR.** Means with standard error from three experiments are represented by a log2 fold change in expression levels normalized to *ewg*, *Appl* and *actin* genes.
